# The Association between MTHFR Gene Polymorphisms and Hepatocellular Carcinoma Risk: A Meta-Analysis

**DOI:** 10.1371/journal.pone.0056070

**Published:** 2013-02-14

**Authors:** Xue Qin, Qiliu Peng, Zhiping Chen, Yan Deng, Shan Huang, Juanjuan Xu, Haiwei Li, Shan Li, Jinmin Zhao

**Affiliations:** 1 Department of Clinical Laboratory, First Affiliated Hospital of Guangxi Medical University, Nanning, Guangxi, China; 2 Department of Occupational Health and Environmental Health, School of Public Health at Guangxi Medical University, Nanning, Guangxi, China; 3 Department of Orthopedic Trauma Surgery, First Affiliated Hospital of Guangxi Medical University, Nanning, Guangxi, China; 4 Department of Clinical Laboratory, Baise City People’s Hospital, Baise, Guangxi, China; Dana-Farber Cancer Institute, United States of America

## Abstract

**Background:**

The association between methylenetetrahydrofolate reductase (MTHFR) gene polymorphisms and hepatocellular carcinoma (HCC) risk was inconsistent and underpowered. To clarify the effects of MTHFR gene polymorphisms on the risk of HCC, a meta-analysis of all available studies relating C677T and/or A1298C polymorphisms of MTHFR gene to the risk of HCC was conducted.

**Methods:**

The authors searched PubMed, EMBASE, Cochrane Library, Web of Science, and Chinese Biomedical Literature database (CBM) for the period up to July 2012. Data were extracted by two independent authors and pooled odds ratio (OR) with 95% confidence interval (CI) was calculated. Metaregression and subgroup analyses were performed to identify the source of heterogeneity.

**Results:**

Finally, 12 studies with 2,351 cases and 4,091 controls were included for C677T polymorphism and 6 studies with 1,333 cases and 1,878 controls were included for A1298C polymorphism. With respect to A1298C polymorphism, significantly decreased HCC risk was found in the overall population (CC vs. AA: OR = 0.660, 95%CI 0.460–0.946, P = 0.024; recessive model: OR = 0.667, 95%CI = 0.470–0.948, P = 0.024). In subgroup analyses, significantly decreased HCC risk was found in Asian population (CC vs. AA: OR = 0.647, 95%CI = 0.435–0.963; P = 0.032) and population-based studies (CC vs. AA: OR = 0.519, 95%CI = 0.327–0.823; P = 0.005). With respect to C677T polymorphism, no significant association with HCC risk was demonstrated in overall and stratified analyses.

**Conclusions:**

We concluded that MTHFR A1298C polymorphism may play a protective role in the carcinogenesis of HCC. Further large and well-designed studies are needed to confirm this association.

## Introduction

Hepatocellular carcinoma (HCC) is the fifth most common cancer and the third leading cause of cancer-related death worldwide, which is still a global health challenge [1], [2]. The mechanism of its carcinogenesis, like other cancers, still remains unclear. Folate is a form of the water-soluble vitamin B9. It is necessary for the production and maintenance of new cells and is involved in DNA methylation, DNA synthesis and DNA repair [3]. Some studies have indicated that folate deficiency could inﬂuence cancer risk [4], [5]. Methylenete trahydrofolate reductase (MTHFR) is a key enzyme for intracellular folate homeostasis and metabolism. It catalyses the irreversible conversion of 5,10-methylenetetrahydrofolate to 5-methyltetrahydrofolate, which is the primary circulating form of folate and provides methyl groups for the methylation of homocysteine to methionine [6]. Altered MTHFR enzyme activity has been linked to the development of cancer [7], [8], [9].

The MTHFR gene is located at chromosome 1p36.3 and is 2.2 kb in length with a total of 11 exons [10]. There are at least 247 single nucleotide polymorphisms (SNPs) in the MTHFR gene, reported in the dbSNP database (http://www.ncbi.nlm.nih.gov/snp/). However, only two common polymorphisms, C677T (rs1801133) and A1298C (rs1801131), have been extensively investigated. For the MTHFR C677T polymorphism, a C to T transition at nucleotide position 677 in exon 4 generates an alanine (Ala) to valine (Val) change at amino acid 222 (Ala222Val). This substitution lies at the binding site for the ﬂavin adenine dinucleotide, an important cofactor for MTHFR [11]. As a result, carriers of the MTHFR 677TT genotype possess a thermolabile enzyme of reduced activity [12], which results in decreased folate concentration and increased homocysteine level in the serum [13]. Another polymorphism in MTHFR, A to C transversion at nucleotide 1298 (A1298C), results in an amino acid substitution of glutamic acid for alanine (Ala) at codon 429 (Glu429Ala), which may also induce decreased activity of MTHFR [14]. Hence, it is biologically reasonable to hypothesize a potential relationship between MTHFR polymorphisms and HCC risk.

A number of studies have been conducted to investigate the association between MTHFR C677T and/or A1298C polymorphisms and HCC risk, but the results are somewhat controversial and underpowered. With respect to C677T polymorphism, a meta-analysis by Jin et al. [15] found that MTHFR C677T polymorphism was associated with an increased HCC risk in an overdominant model, however, they only included 10 eligible studies in the meta-analysis, which make their conclusions questionable. With respect to A1298C polymorphism, to the best of our knowledge, no meta-analyses on this issue have ever appeared. To derive a more precise estimation of the relationship between MTHFR polymorphisms and HCC risk, we conducted a meta-analysis of all available case–control studies relating the C677T and/or A1298C polymorphisms of the MTHFR gene to the risk of developing HCC.

## Methods

### Search Strategy

This study was performed according to the proposal of Meta-analysis of Observational Studies in Epidemiology group (MOOSE) [16]. A comprehensive search strategy was conducted towards the electronic databases including PubMed, EMBASE, Cochrane Library, Web of Science, and Chinese Biomedical Literature database (CBM), using the search strategy based on combinations of the keywords “hepatocellular carcinoma or HCC” and “methylenetetrahydrofolate reductase, MTHFR, one-carbon metabolism or folate” and “polymorphism, mutation or variant”. The last search was updated on July 01, 2012. Although no language restrictions were applied initially, for the full-text review and final analysis our resources only permitted the review of articles published in English and Chinese. Reference lists of the identified articles were also examined and the literature retrieval was performed in duplication by two independent reviewers (Xue Qin and Qiliu Peng). When multiple publications reported on the same or overlapping data, we chose the most recent or largest population. When a study reported the results on different subpopulations, we treated it as separate studies in the meta-analysis.

### Selection Criteria

We reviewed abstracts of all citations and retrieved studies. The following criteria were used to include published studies: (1) evaluating the association between MTHFR gene polymorphisms and HCC; (2) case-control design; (3) the papers must offer the size of the samples, distribution of alleles, genotypes or other information that can help us infer the results to estimate the odds ratio (ORs) and their 95% confidence intervals (CIs); and (4) studies published in English or Chinese language. Participants could be of any age. Studies were excluded if one of the following existed: (1) the design was based on family or sibling pairs; (2) the genotype frequency was not reported; or (3) there was insufficient information for data extraction.

### Data Extraction

Two investigators (Xue Qin and Qiliu Peng) independently extracted data from the studies included. Data extracted from eligible studies included the first author’s name, publication date, country of origin, ethnicity, genotyping method, matching criteria, source of control, HCC diagnosis, QC when genotyping, total numbers of cases and controls and genotype frequencies of cases and controls. The two investigators checked the data extraction results and reached consensus on all of the data extracted. If different results were generated, they would check the data again and have a discussion to come to an agreement. A third reviewer (Li Shan) was invited to the discussion if disagreement still existed.

### Quality Score Assessment

The quality of the eligible studies was independently assessed by two investigators (Xue Qin and Qiliu Peng) according to a set of predefined criteria (Table 1), which was originally proposed by Thakkinstian et al [17]. The revised criteria cover the credibility of controls, the representativeness of cases, specimens of cases determining genotypes, Hardy-Weinberg equilibrium in controls, and total sample size (Table 1). The disagreements between two investigators were resolved by consensus. The total scores ranged from 0 (lowest) to 15 (highest), and studies with scores ≥10 were classified as high-quality studies, whereas studies with scores <10 were considered as low-quality studies.

**Table 1:** Scale for quality assessment.

Criteria	Score
Representativeness of cases	
Selected from population or cancer registry	3
Selected from hospital	2
Selected from pathology archives, butwithout description	1
Not described	0
Credibility of controls	
Population-based	3
Blood donors or volunteers	2
Hospital-based (cancer-free patients)	1
Not described	0
Specimens of cases determining genotypes	
White blood cells or normal tissues	3
Tumor tissues or exfoliated cells of tissue	0
Hardy-Weinberg equilibrium in controls	
Hardy-Weinberg equilibrium	3
Hardy-Weinberg disequilibrium	0
Total sample size	
≥1000	3
≥400 but <1000	2
≥200 but <400	1
<200	0

### Statistical Analysis

Summary odds ratios (ORs) and corresponding 95% confidence intervals (CIs) were estimated for each polymorphism in different comparison models, including additive genetic models, recessive genetic model, and dominant genetic model.

The *Q* test and *I^2^* statistics were used to assess the statistical heterogeneity among studies [18], [19]. If the result of the *Q* test was *P_Q_* <0.1 or *I^2^*≥50%, indicating the presence of heterogeneity, a random-effects model (the DerSimonian and Laird method) was used to estimate the summary ORs [20]; otherwise, when the result of the *Q* test was *P_Q_* ≥0.1 and *I^2^*<50%, indicating the absence of heterogeneity, the fixed-effects model (the Mantel–Haenszel method) was used [21]. To explore the sources of heterogeneity among studies, we performed logistic metaregression and subgroup analyses. The following study characteristics were included as covariates in the metaregression analysis: genotyping methods (PCR-RFLP versus not PCR-RFLP), ethnicity (Caucasian population versus Asian population), quality score (high quality studies versus low quality studies), source of controls (Hospital-based versus Population-based), QC when genotyping (Yes versus no), and HCC diagnosis (pathologically or histologically confirmed versus other diagnosis criteria). Subgroup analyses were conducted by stratification of ethnicity and source of controls. Galbraith plots analysis was performed for further exploration of the heterogeneity.

Sensitivity analysis was performed by sequential omission of individual studies. For each polymorphism, publication bias was evaluated using a funnel plot and Egger’s regression asymmetry test [22]. If publication bias existed, the Duval and Tweedie non-parametric “trim and fill” method was used to adjust for it [23]. The distribution of the genotypes in the control population was tested for Hardy-Weinberg equilibrium using a goodness-of-fit Chi-square test. All analyses were performed using Stata software, version 10.0 (Stata Corp., College Station, TX). All *p* values were two-sided. To ensure the reliability and the accuracy of the results, two authors entered the data into the statistical software programs independently with the same results.

## Results

### Study Characteristics

**Figure 1:**
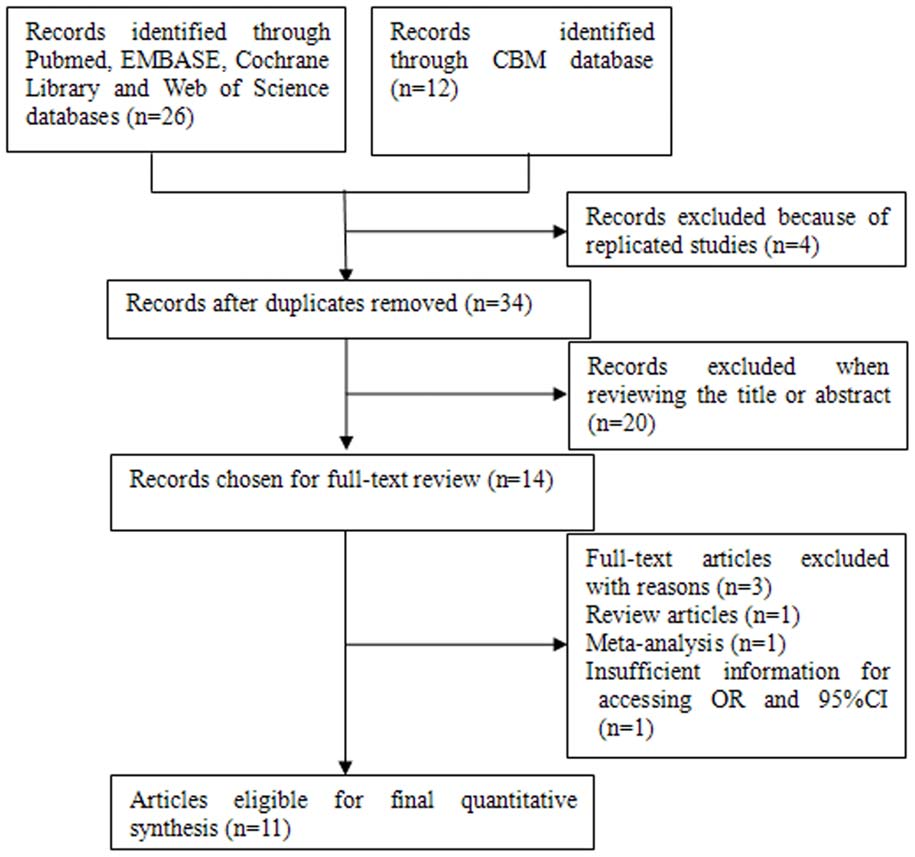
Flow diagram of included studies for this meta-analysis.

Based on the search criteria, 14 studies relevant to the role of MTHFR gene polymorphisms on HCC susceptibility were identified. Three of these articles were excluded: one of these articles was a review [24], one was a meta-analysis [15], and one did not provide allele or genotyping data [25]. Manual search of references cited in the published studies did not reveal any additional articles. As a result, a total of 11 relevant studies including 9 English articles [26]–[34] and 2 Chinese papers (one was a dissertation of postgraduate student) [35], [36] met the inclusion criteria for the meta-analysis (Figure 1). Among them, one of the eligible studies contained data on two different ethnic groups [30], and we treated it independently. Therefore, a total of 12 separate comparisons were finally included in our meta-analysis. The main characteristics of the studies were presented in Table 2. Among them, six studies evaluated the C677T variant and 6 studies evaluated the C677T and A1298C variants. Therefore, a total of 12 studies including 2,351 cases and 4,091 controls were available for the meta-analysis of C677T polymorphism and 6 studies containing 1,333 cases and 1,878 controls were included for A1298C polymorphism. The sample size in these studies varied considerably, ranging from 150 to 1,051 individuals. Of all the eligible studies, 5 were conducted in Caucasian population, and 7 were in Asians for C677T polymorphism; 5 were conducted in Asians and only one [30] was in Caucasians for A1298C polymorphism. Six studies were population–based and 6 were hospital–based studies. Only 3 articles of all eligible studies used quality control when genotyping and 4 studies in the present meta-analysis did not provide definite criteria for the HCC diagnosis. Several genotyping methods were used, including PCR-RFLP, TaqMan assay, and RT-PCR. The genotype distributions of the controls in two studies were not consistent with HWE for C677T polymorphism [27], [30] and one was not consistent with HWE for A1298C polymorphism [34].

**Table 2:** Characteristics of eligible studies.

First author (Year)	Country	Ethnicity	Sample size (case/control)	Genotyping methods	Matching criteria	Source of control	HCC diagnosis	QC when Genotyping	PI	HWE(*P* value)		Quality scores
										C677T	A1298C	
Saffroy, 2004	France	Caucasian	148/232	PCR-RFLP	Age, gender	PB	HC	No	C677T	0.291	–	10
Ventura 2005	Italy	Caucasian	22/128	PCR-RFLP	Age	PB	NA	No	C677T	**0.003**	–	6
Zhu 2006	China	Asian	508/543	PCR-RFLP	Gender, smoking	HB	PC and HC	Yes	C677T	0.921	–	12
Yang 2007	China	Asian	322/185	PCR-RFLP	Gender, smoking	HB	HC	Yes	C677T, A1298C	0.139	0.769	14
Mu 2007	China	Asian	194/391	PCR-RFLP	Region	PB	PC	No	C677T, A1298C	0.235	0.249	10
Yuan1 2007	China	Asian	247/248	TaqMan Assay	Age, gender	PH	HC	No	C677T, A1298C	**0.033**	0.305	11
Yuan2 2007	America	Caucasian	118/209	TaqMan Assay	Age, gender	PB	HC	No	C677T, A1298C	0.944	0.666	10
Kwak 2008	Korea	Asian	96/201	PCR-RFLP	Age	HB	NA	No	C677T, A1298C	0.234	0.261	10
D’Amico 2009	Italy	Caucasian	94/308	PCR-RFLP	Age, gender	HB	NA	No	C677T	0.207	–	9
Fabris 2009	Italy	Caucasian	65/381	PCR-RFLP	Age, gender	HB	HC	No	C677T	0.521	–	11
Liu 2010	China	Asian	181/624	TaqMan Assay	Region	HB	NA	Yes	C677T	0.189	–	11
Cui 2011	China	Asian	356/641	RT-PCR	Age, Region	PB	PC	No	C677T, A1298C	0.483	**0.003**	10

PI, Polymorphism(s) investigated; HC, Histologically confirmed; PC, Pathologically confirmed; NA, Not available; QC, Quality control; PB, Population–based; HB, Hospital–based; HWE, Hardy–Weinberg equilibrium in control population; PCR–RFLP, Polymerase chain reaction-restriction fragment length polymorphism; RT–PCR, Real time–polymerase chain reaction.

### Meta-analysis Results

**Figure 2:**
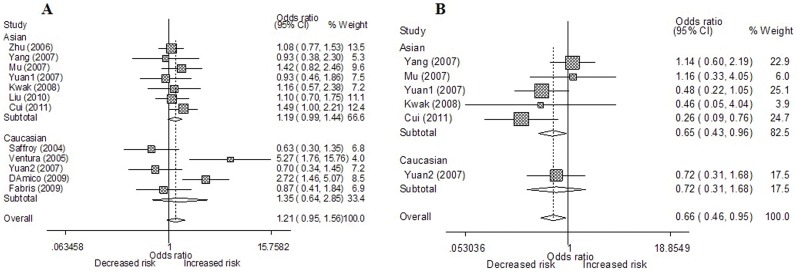
Forest plots of MTHFR gene polymorphisms and HCC risk. A Forest plots of MTHFR C677T polymorphism and HCC risk in subgroup analysis by ethnicity using a random-effect model (contrast TT vs. CC); **B** Forest plots of MTHFR A1298C polymorphism and HCC risk in subgroup analysis by ethnicity using a fixed-effect model (CC vs. AA).

The meta-analysis suggested that the C677T polymorphism was not associated with HCC risk in all genetic models (additive models TT vs. CC and CT vs. CC, recessive model, and dominant model; Table 3) in the overall populations. Moreover, we failed to identify any significant association between the C677T polymorphism and HCC risk in all comparison models in subgroup analyses according to ethnicity and source of controls (Table 3, Figure 2A).

**Table 3:** Meta-analysis of the MTHFR gene polymorphisms on HCC risk.

Comparison	Population	No. of studies	Test of association			Mode	Test of heterogeneity		
			OR	95% CI	*P* Value		*χ^2^*	*P_Q_* Value	*I^2^*
C677T									
TT vs. CC	Overall	12	1.213	0.946–1.555	0.128	R	22.04	0.024	50.1
	Caucasian	5	1.351	0.640–2.853	0.430	R	19.08	0.001	79.0
	Asian	7	1.192	0.988–1.439	0.067	F	2.82	0.831	0.0
	PB	6	1.211	0.777–1.889	0.398	R	13.87	0.016	64.0
	HB	6	1.190	0.956–1.482	0.119	F	8.12	0.150	38.4
CT vs. CC	Overall	12	1.001	0.884–1.134	0.984	F	11.34	0.415	3.0
	Caucasian	5	0.896	0.701–1.146	0.383	F	2.48	0.649	0.0
	Asian	7	1.040	0.900–1.202	0.593	F	7.85	0.250	23.5
	PB	6	1.081	0.902–1.295	0.399	F	7.65	0.177	34.6
	HB	6	0.934	0.787–1.109	0.436	F	2.43	0.787	0.0
TT vs. CT+CC	Overall	12	1.194	0.979–1.457	0.080	R	19.01	0.061	42.1
	Caucasian	5	1.406	0.740–2.672	0.298	R	16.88	0.002	76.3
	Asian	7	1.151	0.987–1.343	0.073	F	1.20	0.977	0.0
	PB	6	1.133	0.792–1.621	0.493	R	11.58	0.041	56.8
	HB	6	1.252	0.981–1.598	0.070	F	7.16	0.209	30.2
TT+CT vs. CC	Overall	12	1.058	0.905–1.237	0.481	R	18.16	0.078	39.4
	Caucasian	5	1.048	0.712–1.542	0.812	R	10.47	0.033	61.8
	Asian	7	1.072	0.935–1.228	0.319	F	7.34	0.290	18.3
	PB	6	1.111	0.837–1.475	0.467	R	12.54	0.028	60.1
	HB	6	1.004	0.856–1.179	0.960	F	5.01	0.415	0.2
A1298C									
CC vs. AA	Overall	6	**0.660**	**0.460**–**0.946**	**0.024**	F	7.19	0.207	30.5
	Caucasian	1	0.720	0.309–1.677	0.446	–	–	–	–
	Asian	5	**0.647**	**0.435**–**0.963**	**0.032**	F	7.20	0.126	44.4
	PB	4	**0.519**	**0.327**–**0.823**	**0.005**	F	3.78	0.286	20.7
	HB	2	1.045	0.567–1.927	0.888	F	0.62	0.432	0.0
AC vs. AA	Overall	6	1.055	0.900–1.236	0.510	F	3.44	0.633	0.0
	Caucasian	1	0.828	0.513–1.336	0.440	–	–	–	–
	Asian	5	1.087	0.919–1.286	0.331	F	2.33	0.676	0.0
	PB	4	1.033	0.860–1.241	0.725	F	1.15	0.756	0.0
	HB	2	1.121	0.817–1.538	0.480	F	2.08	0.149	32.1
CC vs. AC+AA	Overall	6	**0.667**	**0.470**–**0.948**	**0.024**	F	8.13	0.149	38.5
	Caucasian	1	0.780	0.343–1.774	0.554	–	–	–	–
	Asian	5	0.627	0.330–1.192	0.154	R	8.07	0.089	50.4
	PB	4	**0.522**	**0.332**–**0.821**	**0.005**	F	4.34	0.227	30.8
	HB	2	1.054	0.583–1.903	0.863	F	0.82	0.365	0.0
CC+AC vs. AA	Overall	6	0.995	0.855–1.159	0.953	F	2.73	0.742	0.0
	Caucasian	1	0.808	0.513–1.270	0.355	–	–	–	–
	Asian	5	1.023	0.870–1.202	0.788	F	1.80	0.772	0.0
	PB	4	0.958	0.803–1.143	0.634	F	0.67	0.881	0.0
	HB	2	1.113	0.823–1.506	0.485	F	1.33	0.249	24.8

OR, odds ratio; CI, confidence intervals; R, random effects model; F, fixed effects model; PB, Population–based; HB, Hospital–based.

For the A1298C polymorphism, significant decreased HCC risk was found in additive model CC vs. AA (OR = 0.660, 95%CI 0.460–0.946, *P* = 0.024; *I^2^* = 30.5 and *P_Q_*_ = _0.207 for heterogeneity; Figure 2B) and recessive model CC vs. AC+AA (OR = 0.667, 95%CI = 0.470–0.948, *P* = 0.024; *I^2^* = 38.5 and *P_Q_*_ = _0.149 for heterogeneity) in the overall populations. Subgroup analysis stratified by source of controls showed that the A1298C polymorphism was associated with a significantly decreased HCC risk among population-based studies for additive model CC vs. AA (OR = 0.519, 95%CI = 0.327–0.823, *P* = 0.005; *I^2^* = 20.7 and *P_Q_*_ = _0.286 heterogeneity) and recessive model CC vs. AC+AA (OR = 0.522, 95%CI = 0.332–0.821, *P* = 0.005; *I^2^* = 30.8 and *P_Q_*_ = _0.227 for heterogeneity). When stratified by ethnicity, significant decreased HCC risk was also found in Asians in additive model CC vs. AA (OR = 0.647, 95%CI = 0.435–0.963, *P* = 0.032; *I^2^* = 44.4 and *P_Q_*_ = _0.126 for heterogeneity) but not in recessive model CC vs. AC+AA (OR = 0.627, 95%CI = 0.330–1.192, *P* = 0.154; *I^2^* = 50.4 and *P_Q_*_ = _0.089 for heterogeneity). Interestingly, when we excluded the study by Yang et al [36]. which was shown as an outlier in our Galbraith plots analysis, the summary OR of recessive model CC vs. AC+AA in Asian population reached significance (OR = 0.441, 95%CI: 0.259–0.750, *P* = 0.003; *P_Q_* = 0.342 and *I^2^* = 10.2 for heterogeneity).

### Heterogeneity Analysis

For the C677T polymorphism, the *I^2^* values of heterogeneity were greater than 50% and the *P_Q_* values were lower than 0.10 in additive model TT vs. CC, recessive model TT vs. CT+CC, and dominant model TT+CT vs. CC in the overall populations, which indicated statistically significant heterogeneity among studies. To explore the sources of heterogeneity, we performed metaregression and subgroup analyses. Metaregression analysis of data showed that the ethnicity and source of controls were the major sources which contributed to heterogeneity. The ethnicity and source of controls were both positively associated with the ORs in additive model TT vs. CC (regression coefficient = 0.588, 95%CI: 0.072–1.104, *p* = 0.026 for ethnicity and regression coefficient = 1.510, 95%CI: 0.634–2.385, *p* = 0.001 for source of controls, respectively), recessive model TT vs. CT+CC (regression coefficient = 0.439, 95%CI: 0.124–0.802, *p* = 0.041 for ethnicity and regression coefficient = 1.231, 95%CI: 0.459–2.004, *p* = 0.002 for source of controls, respectively), and dominant model TT+CT vs. CC (regression coefficient = 0.482, 95%CI: 0.135–0.826, *p* = 0.006 for ethnicity and regression coefficient = 0.917, 95%CI: 0.265–1.569, *p* = 0.006 for source of controls, respectively). The Genotyping methods, HCC diagnosis, QC when genotyping, and Quality scores were not effect modifiers. Subsequently, we performed subgroup analyses stratified by ethnicity and source of controls. However, heterogeneity still existed among Caucasians and population-based studies in all the above three genetic comparison models (table 3).

**Figure 3:**
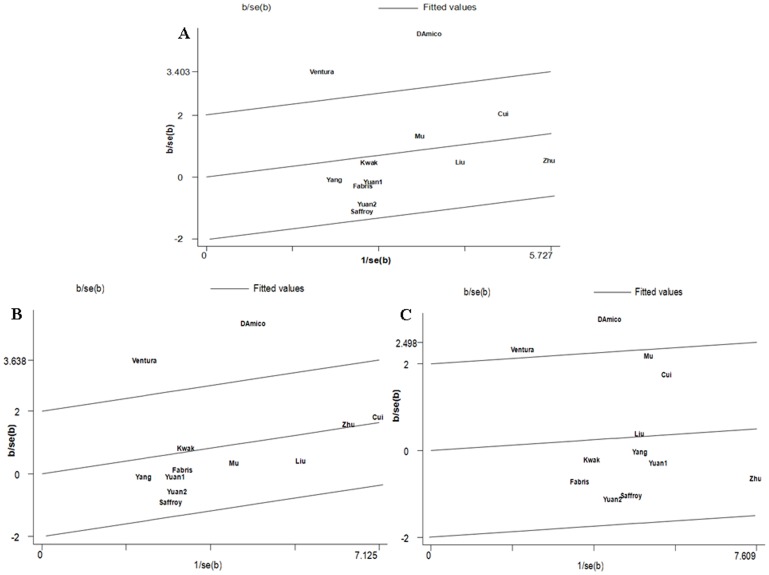
Galbraith plots of C677T polymorphism and HCC risk in different contrast models. **A** The studies of Ventura et al., D’Amico et al. were outliers in the contrast TT vs. CC. **B** The studies of Ventura et al., D’Amico et al. were outliers in the recessive model TT vs. CT+CC. **C** The studies of Ventura et al., D’Amico et al. were outliers in the dominant model TT+CT vs. CC.

To further investigate the heterogeneity, we performed Galbraith plots analysis to identify the outliers which might contribute to the heterogeneity. Our results showed that Ventura et al. [27] and D’Amico et al. [32] were outliers in additive model TT vs. CC, recessive model TT vs. CT+CC, and dominant model TT+CT vs. CC model for C677T polymorphism (Figure 3). All *I^2^* values decreased obviously and *P_Q_* values were greater than 0.10 after excluding the studies of Ventura et al. [27] and D’Amico et al. [32] in all genetic comparison models in the overall populations (additive model TT vs. CC: *P_Q_* = 0.596, *I^2^* = 0.0; recessive model TT vs. CT+CC: *P_Q_* = 0.915, *I^2^* = 0.0; dominant model TT+CT vs. CC: *P_Q_* = 0.251, *I^2^* = 20.9 ), Caucasians (additive model TT vs. CC: *P_Q_* = 0.831, *I^2^* = 0.0; recessive model TT vs. CT+CC: *P_Q_* = 0.740, *I^2^* = 0.0; dominant model TT+CT vs. CC: *P_Q_* = 0.986, *I^2^* = 0.0), and population-based studies (additive model TT vs. CC: *P_Q_* = 0.418, *I^2^* = 41.0; recessive model TT vs. CT+CC: *P_Q_* = 0.520, *I^2^* = 0.0; dominant model TT+CT vs. CC: *P_Q_* = 0.149, *I^2^* = 38.2). The significance of the summary ORs for the C677T polymorphism in different comparison models in the overall population and subgroup analyses were not inﬂuenced by omitting the two studies.

**Figure 4:**
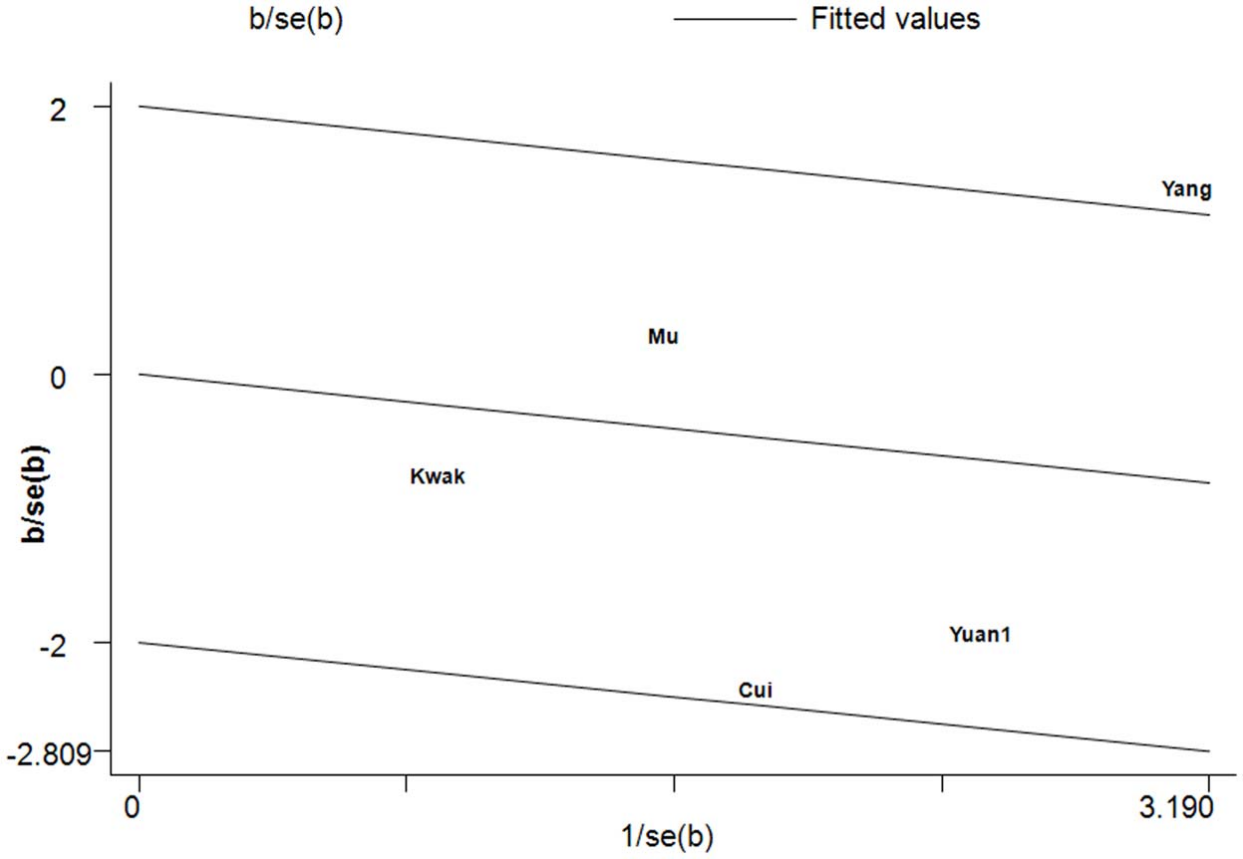
Galbraith plots of A1298C polymorphism and HCC risk in Asians, The study of Yang et al. was the outlier in recessive model CC vs. AC+AA.

For the A1298C polymorphism, there was no statistical significant heterogeneity in all comparison models in the overall populations. Subgroup analysis by ethnicity and source of controls also indicated no significant heterogeneity in all comparison models except the recessive model CC vs. AC+AA in Asians (*P_Q_* = 0.089, *I^2^* = 50.4; Table 3). Galbraith plots analysis showed that the study Yang et al. [36] was the outlier (Figure 4). The *I^2^* value decreased lower than 50% and *P_Q_* values were greater than 0.10 after excluding the study of Yang et al. (*P_Q_* = 0.342, *I^2^* = 10.2). Interestingly, the summary OR of recessive model CC vs. AC+AA in Asians reached significance after omitting this study (OR = 0.441, 95%CI: 0.259–0.750, *P* = 0.003).

### Sensitivity Analysis

A single study involved in the meta-analysis was deleted each time to reflect the influence of the individual data-set to the pooled ORs, and the corresponding pooled ORs were not materially altered (data not shown), indicating that our results were statistically robust. Although the genotype distribution in two studies of C677T polymorphism [27], [30] and one study of A1298C polymorphism [34] was not in accordance with HWE, the corresponding pooled ORs were not qualitatively altered with or without including these studies.

### Publication Bias

**Figure 5:**
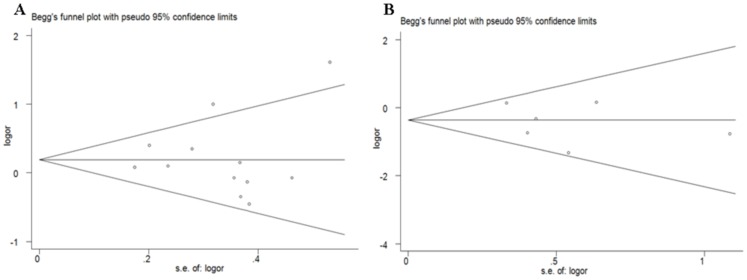
Funnel plot analysis to detect publication bias. Each point represents a separate study for the indicated association. **A** Funnel plot for contrast TT vs. CC of C677T polymorphism in overall analysis; **B** Funnel plot for allele contrast CC vs. AA of A1298C polymorphism in overall analysis.

Begg’s funnel plot and Egger’s test were performed to assess the publication bias of literatures in all comparison models. The shape of the funnel plot did not reveal any evidence of obvious asymmetry (Figure 5). Then, the Egger’s test was used to provide statistical evidence of funnel plot symmetry. The results still did not suggest any evidence of publication bias in C677T (*P* = 0.900 for TT vs. CC; *P* = 0.804 for CT vs. CC; *P* = 0.834 for recessive model TT vs. CT+CC; and *P* = 0.365 for dominant model TT+CT vs. CC) and A1298C (*P* = 0.508 for CC vs. AA; *P* = 0.717 for AC vs. CC; *P* = 0.458 for recessive model CC vs. AC+AA; and *P* = 0.409 for dominant model AC+CC vs. AA) polymorphisms.

## Discussion

The folate metabolism pathway plays an important role in DNA synthesis and DNA methylation which is directed by purine and pyrimidine synthesis; folate deficiency causes uracil misincorporation into DNA with subsequent chromosome breaks [37]. MTHFR is a key enzyme in the folate metabolism pathway [38]. Two common variations in the MTHFR gene, C677T and A1298C, were associated with reduced MTHFR activity. It was reported that homozygotes (TT) and heterozygotes (CT) for C677T have, respectively, 30% and 65% of the enzyme activity compared with those whose genotype is homozygotes (CC), whereas homozygotes (CC) for A1298C have only 60% of the normal enzyme activity [12], [39]. Decreased MTHFR activity may lead to an alteration of normal intracellular distribution of folate substrates [40], and result in tumor susceptibility. This hypothesis was confirmed by our meta-analysis.

Our meta-analysis results showed that individuals with the 1298CC genotype had a reduced risk of HCC compared to those with the 1298AA genotype, especially among the Asian population. However, no association was detected among the Caucasian population. In addition, our data also showed a decreased HCC risk under the recessive genetic model (CC vs. AC+AA) in the overall populations. When we excluded the study of Yang et al. [36] which was shown as an outlier in Galbraith plots analysis, a statistically significant decreased HCC risk was also found in Asian population but not in Caucasians under the recessive genetic model. Actually, it might not be uncommon for the same polymorphism play different roles in cancer susceptibility among different ethnic populations. In Caucasians, the differences in genetic backgrounds and the environment they lived in may inﬂuence the association between the MTHFR A1298C polymorphism and the risk for HCC. In addition, the limited number of studies also makes the results from subgroup analysis by ethnicity less reliable. Thus, our results should be interpreted with caution.

In the subgroup analysis based on source of controls, significantly decreased HCC risk was found in MTHFR 1298CC genotype carriers in the population-based studies but not in hospital-based studies. This reason may be that the hospital-based studies have a high risk of producing unreliable results because hospital-based controls may not always be truly representative of the general population. Therefore, a methodologically preferable design, such as using a proper and representative population-based study, is crucial to avoid selection bias.

With respect to C677T polymorphism, 12 studies were found in our meta-analysis. Contrary to the previous findings made by Jin et al. [15], our results showed that MTHFR C677T polymorphism was not associated with HCC risk not only in the overall population but also in the subgroup analyses stratified by ethnicity and source of controls. This is most probably because of the relatively small sample size of the previous meta-analysis. The meta-analysis of Jin et al. included only 10 studies for evaluating the association between MTHFR C677T polymorphism and HCC risk and may have insufficient statistical power to detect a true effect or may have generated a ﬂuctuated risk estimate. Therefore, a meta-analysis with relatively larger sample size (including original studies as many as possible) is crucial to avoid selection bias in such genotype association studies.

Heterogeneity analysis of C677T polymorphism suggested significant heterogeneity in additive model TT vs. CC, recessive model TT vs. CT+CC, and dominant model TT+CT vs. CC in the overall populations. To explore the sources of heterogeneity, we performed metaregression and subgroup analyses. Metaregression analysis of data showed that the ethnicity and source of controls but not Genotyping methods, HCC diagnosis, QC when genotyping, and Quality scores might substantially inﬂuence the initial heterogeneity. Subgroup analyses by ethnicity and source of controls indicated that heterogeneity still existed in Caucasians and population-based studies in all the above three genetic comparison models. To further investigate the heterogeneity, Galbraith plots analysis was performed to identify the outliers which might contribute most to the heterogeneity. Our results showed that the studies of Ventura et al. [27] and D’Amico et al. [32] were outliers of the above three genetic comparison models (Figure 3). All *I^2^* values decreased lower than 50% and *P_Q_* values were greater than 0.10 after excluding the studies of Ventura et al. [27] and D’Amico et al. [32] in all genetic comparison models in the overall populations, Caucasians and the population-based studies. In addition, the summary ORs for the C677T polymorphism in different comparison models in the overall population and subgroup analyses were not material change by omitting the two studies, indicating that our results were robust and reliable. The results indicated that the two studies might be the major source of the heterogeneity for the C677T polymorphism.

Significant heterogeneity was found in the recessive model CC vs. AC+AA (*I^2^* = 50.4%, *P_Q_* = 0.089) for the A1298C polymorphism in Asian populations. Galbraith plots analysis showed that the study Yang et al. [36] was the outlier (Figure 4). The *I^2^* value decreased lower than 50% and *P_Q_* values were greater than 0.10 after excluding this study (*P_Q_* = 0.342, *I^2^* = 10.2). Interestingly, the pooled OR of recessive model in Asians reached significance after omitting this study (OR = 0.441, 95%CI: 0.259–0.750, *P* = 0.003). The results indicated that the study of Yang et al. [36] was the main source of heterogeneity for the A1298C polymorphism.

This meta-analysis should be interpreted with caution at the present time because of some limitations. First, the overall outcomes were based on individual unadjusted ORs, whereas a more precise evaluation should be adjusted by potentially suspected factors, including age, gender, smoking status, and environmental factors. In some studies, individuals who were unmatched by age and gender later developed HCC within the age range in the control group. The results would hence underestimate the OR association with the genotype. Second, the controls were not uniformly defined. Although most of the controls were selected mainly from healthy populations, some had benign disease such as liver cirrhosis, HBsAg positive subjects and so on. Therefore, non-differential misclassification bias was possible because these studies may have included the control groups who have different risks of developing HCC. Third, the number of studies included in this study for A1298C polymorphism was relatively small and there was only one study in the Caucasian group [29], which leaded to low statistical power. Forth, bias may result from the fact that unpublished data, as well as papers published in languages other than English and Chinese, were not included. Fifth, all of the studies included in the meta-analysis were performed in Asian and Caucasian populations; further studies are needed in other ethnic groups in order to capture the full range of possible ethnic differences in MTHFR polymorphisms.

In summary, the present meta-analyses did not support a prominent association between MTHFR C677T polymorphism and HCC risk. The A1298C polymorphism might be associated with decreased HCC risk in Asian populations based on current published studies. However, it is necessary to conduct large sample studies using standardized unbiased genotyping methods, homogeneous HCC patients and well matched controls. Moreover, gene–gene and gene–environment interactions should also be considered in the analysis. Such studies taking these factors into account may eventually lead to better, comprehensive understanding of the association between the MTHFR polymorphisms and HCC risk.

## Supporting Information

Figure S1 **Flow diagram of included studies for this meta-analysis.**
(TIF)

Table S1 **Checklist.**
(DOC)
