# Supplementary figures and images for: The Association between MTHFR Gene Polymorphisms and Hepatocellular Carcinoma Risk: A Meta-Analysis
Source: PLoS One. 2013 Feb 14;8(2):e56070. doi: 10.1371/journal.pone.0056070 (PMC3573065; doi:10.1371/journal.pone.0056070)

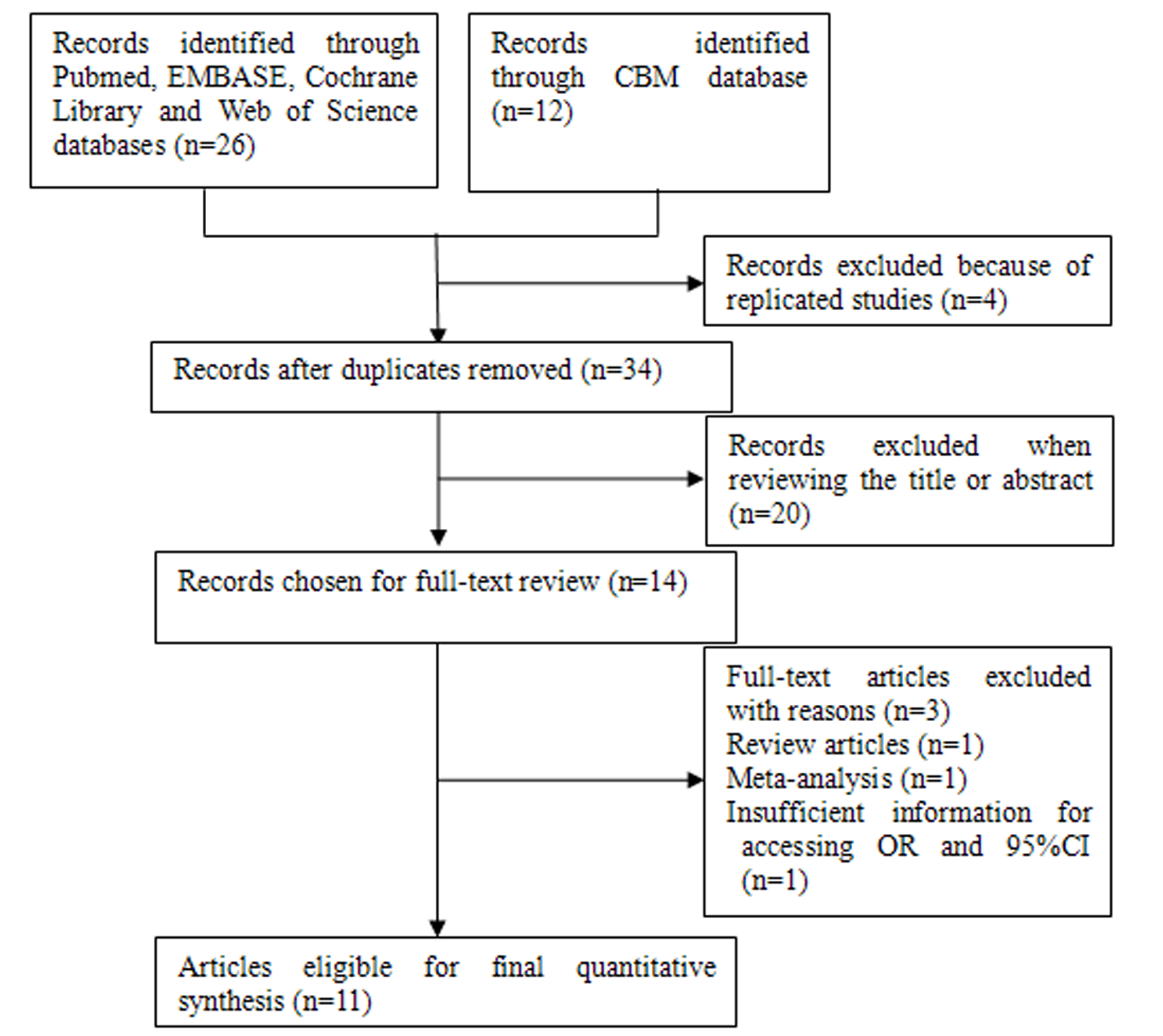

Supplement: Figure S1 — Flow diagram of included studies for this meta-analysis. (TIF) [file pone.0056070.s001.tif]
